# Varicella Zoster Infection: A Rare Cause of Abdominal Pain Mimicking Acute Abdomen

**DOI:** 10.4021/jocmr2009.09.1259

**Published:** 2009-10-16

**Authors:** Deniz Olmez, Alper Boz, Nazif Erkan

**Affiliations:** a Izmir Bozyaka Training and Research Hospital, Department of Anaesthesiology, Bozyaka, Izmir, Turkey; b Izmir Bozyaka Training and Research Hospital, Department of Surgery, Bozyaka, Izmir, Turkey

## Abstract

Varicella zoster is an acute viral infection that results from reactivation of a latent varicella zoster virus. It usually occurs in adult population and immune compromised patients. It rarely occurs in healthy children. Here we present a 14 years old male with varicella zoster that had abdominal pain mimicking acute abdomen to alert others who are consulted for the differentiation of acute abdomen and others who may be consulted for pain management.

**Keywords:**

Varicella zoster; Abdominal pain

## Introduction

Varicella zoster is an acute viral infection that results from reactivation of a latent varicella zoster virus. The goal of intervention is to reduce associated pain and discomfort. A prompt diagnosis and appropriate management occur with greater frequency in the adult population. It rarely occurs in otherwise normal children [1]. Varicella zoster virus is known to cause varicella in children and reactivate years later as shingles. Varicella zoster infection usually is a minor illness, but can result in serious life threatening complications in previously healthy patients. Frequent complications include bacterial skin infections, pneumonitis, complications affecting the central nervous system and hepatitis [2,3]. It sometimes mimics acute abdomen especially before the appearance of skin rashes [4-8]. In general surgery clinics, varicella zoster cases are rarely described. In this study, we present an atypical case of varicella zoster with abdominal pain mimicking acute abdomen, and also the literature review.

## Case Report

A 14-year-old male child had been admitted to emergency department of our hospital. He was suffering from a severe, generalized abdominal pain. He had nausea and vomiting accompanying abdominal pain. The patient could not identify a location on his abdomen where the pain was most severe. On physical examination, there was abdominal rigidity, muscle guarding and rebound tenderness. Laboratory tests were non specific. Chest x-ray, abdominal ultrasonography and computerized tomography were normal. We suspected peritonitis. During the two days of follow-up period, the abdominal pain was localized on the left lower quadrant. So, we thought it could be an intestinal tractus disease such as diverticulitis, regional enteritis, etc. No analgesics were given for pain due to suspicion of acute abdomen. In control abdominal ultrasonography, any emergent pathology was not found and the intensity of abdominal pain was decreased. Then the patient was discharged home. In control period, he returned demonstrating the maculopapular lesions of skin over left inguinal region 4 days later. The patient was examined by a dermatologist and it was diagnosed as varicella zoster. Varicella zoster was not diagnosed until he developed a maculopapular rash over left inguinal region 1 week after the onset of pain. Acyclovir was given orally and pomad was applied over the skin lesions. The patients abdominal pain resolved over a few days.

## Discussion

Varicella zoster results from reactivation of the varicella-zoster virus in cells of the dorsal root ganglia after chickenpox. The skin lesions begin as a maculopapular rash that follows a dermatomal distribution. In the absence of cutaneous eruption, visceral zoster is difficult to diagnose [7].

The severe pain of visceral zoster has long been recognized. Visceral pain from herpes zoster can be misdiagnosed as an acute abdomen leading to unnecessary surgical exploration [4]. In one series of 121 negative emergency laparotomies, 3 occurred in patients with varicella zoster virus.

Some relatively common diseases must be considered in the differential diagnosis of acute abdominal pain. The pain can be confusing, particularly if the nerves in the right lower quadrant are involved in a patient whose appendix has not been removed. When our patient admitted to hospital, he had abdominal pain mimicking acute abdomen like appendicitis. Another disease in the differential diagnosis is Meckels diverticulitis, in which abdominal pain is usually in the left lower abdomen but can be anywhere. Elevated white blood cell count presented. Peritonitis, urinary tract infection, gastroenteritis, pneumonia were considered in the differential diagnosis.

The patient was not given analgesic. Recent surveys suggest that many physicians believe conservative administration of pain medication does not interfere with diagnosis and treatment of patients with acute abdominal pain [9,10]. Despite this recognition, the difference between understanding and practice remains large and abdominal pain is often under-treated as it had happened in our case.

In the absence of rash, the diagnosis can be made using polymerase chain reaction (PCR) analysis of blood or fecal samples [11]. Although PCR is the most sensitive method, it is still very expensive and not widely available. Antiviral treatment is usually delayed until cutaneous signs of the infection appear.

In summary, varicella zoster should be suspected in children who have acute abdominal pain but no characteristic skin eruptions. We report this patient to alert others who are consulted for the differentiation of acute abdomen and others who may be consulted for pain management.
